# Fusarium wilt constrains mungbean yield due to reduction in source availability

**DOI:** 10.1093/aobpla/plae021

**Published:** 2024-04-09

**Authors:** Shanice Van Haeften, Yichen Kang, Caitlin Dudley, Andries Potgieter, Hannah Robinson, Eric Dinglasan, Kylie Wenham, Thomas Noble, Lisa Kelly, Colin A Douglas, Lee Hickey, Millicent R Smith

**Affiliations:** Queensland Alliance for Agriculture and Food Innovation, The University of Queensland, QLD 4067, Australia; Queensland Alliance for Agriculture and Food Innovation, The University of Queensland, QLD 4067, Australia; Queensland Alliance for Agriculture and Food Innovation, The University of Queensland, QLD 4067, Australia; Queensland Alliance for Agriculture and Food Innovation, The University of Queensland, QLD 4067, Australia; Queensland Alliance for Agriculture and Food Innovation, The University of Queensland, QLD 4067, Australia; Queensland Alliance for Agriculture and Food Innovation, The University of Queensland, QLD 4067, Australia; Queensland Alliance for Agriculture and Food Innovation, The University of Queensland, QLD 4067, Australia; Department of Agriculture and Fisheries Queensland, QLD 4370, Australia; Department of Agriculture and Fisheries Queensland, QLD 4370, Australia; Department of Agriculture and Fisheries Queensland, QLD 4370, Australia; Queensland Alliance for Agriculture and Food Innovation, The University of Queensland, QLD 4067, Australia; Queensland Alliance for Agriculture and Food Innovation, The University of Queensland, QLD 4067, Australia; School of Agriculture and Food Sustainability, The University of Queensland, QLD 4343, Australia

**Keywords:** *Fusarium oxysporum*, morphology; phenology, seed yield, *Vigna radiata*

## Abstract

Mungbean is an important source of plant protein for consumers and a high-value export crop for growers across Asia, Australia and Africa. However, many commercial cultivars are highly vulnerable to biotic stresses, which rapidly reduce yield within the season. *Fusarium oxysporum* is a soil-borne pathogen that is a growing concern for mungbean growers globally. This pathogen causes Fusarium wilt by infecting the root system of the plant resulting in devastating yield reductions. To understand the impact of *Fusarium* on mungbean development and productivity and to identify tolerant genotypes, a panel of 23 diverse accessions was studied. Field trials conducted in 2016 and 2021 in Warwick, Queensland, Australia under rainfed conditions investigated the variation in phenology, canopy and yield component traits under disease and disease-free conditions. Analyses revealed a high degree of genetic variation for all traits. By comparing the performance of these traits across these two environments, we identified key traits that underpin yield under disease and disease-free conditions. Aboveground biomass components at 50 % flowering were identified as significant drivers of yield development under disease-free conditions and when impacted by *Fusarium* resulted in up to 96 % yield reduction. Additionally, eight genotypes were identified to be tolerant to *Fusarium*. These genotypes were found to display differing phenological and morphological behaviours, thereby demonstrating the potential to breed tolerant lines with a range of diverse trait variations. The identification of tolerant genotypes that sustain yield under disease pressure may be exploited in crop improvement programs.

## Teaser

Stem biomass and HI are key traits underpinning yield in both the presence and absence of Fusarium wilt. *Fusarium* reduced biomass traits, especially pre-flowering, limiting source availability and consequently yield. Diverse lines tolerant to Fusarium wilt were identified, representing valuable donor sources for mungbean improvement.

## Introduction

Mungbean is a significant crop worldwide, occupying over 6 million hectares across tropical and subtropical regions (Kim *et al.* 2015). Due to its short duration (55–70 days), suitability within intercropping systems, low input requirements and being a rich source of protein, mungbean production has substantially increased over the past decade (Yimram *et al.* 2009; Ilyas *et al.* 2018). However, expansion is constrained due to low yield potential and reliability. This is a consequence of vulnerability to abiotic and biotic stresses that contributes to poor yield stability across environments and production systems (Van Haeften *et al.* 2023). The average production of mungbean for growers worldwide currently varies between 0.3 and 1.5 t ha^−1^ depending on the environmental conditions (ABARES 2020; Sequeros *et al.* 2021). These yield values fall substantially short compared to yield recorded in research plots (Chauhan *et al.* 2017; Rachaputi *et al.* 2019) and predictions from crop modelling programs (i.e. Agricultural Production Systems Simulator (APSIM)) (Chauhan *et al.* 2010; Chauhan and Williams 2018). Therefore, enhancing seed yield productivity and reliability stands as a critical objective within global mungbean breeding programmes (Lambrides and Godwin 2007; Nair *et al.* 2020).

Current global commercial mungbean cultivars have a narrow genetic base (Gupta *et al.* 2013) as they have been bred from a small number of highly related breeding lines (Gupta *et al.* 2004). This has led to many commercial cultivars being highly vulnerable to a range of biotic stresses. Fusarium wilt, caused by *Fusarium oxysporum* species, was formerly a minor disease but is now becoming an increasing issue impacting mungbean yield globally (Kelly 2018; Sun *et al.* 2019). In Australia, disease incidence and severity has substantially increased over the last few years. For instance, up to 80 % yield losses have been reported, and in the 2020–21 season, Fusarium wilt caused approximately $4.8 million in losses to the industry (Kelly 2018; GRDC 2022). *Fusarium oxysporum* can infect mungbean crops throughout all developmental stages and initially infects the plant by entering the epidermis of the root tissue and causes root decay. Once the crop is infected, the pathogen spreads through the vascular tissue and colonizes xylem vessels which results in vessel clogging (Di *et al.* 2016; Singh *et al.* 2017). Symptoms can include leaf chlorosis or necrosis, stunting, defoliation, as well as overall death of the plant (Pottorff *et al.* 2012). Fusarium wilt can cause significant yield losses ranging from 30 % to 100 % (Gentry 2010; Kelly 2018; Sun *et al.* 2020). Agronomic management of Fusarium wilt is challenging due to the persistence of *F. oxysporum* in soil for long periods and ease of transmissibility. Thus, breeding for genetic resistance is one avenue to overcome the devastating effects of this disease (Zhu *et al.* 2017; Pegg *et al.* 2019). Until now, most mungbean studies have examined the diversity of strains of *Fusarium* as well as the effects of biological or chemical control (Choudhary *et al.* 2017; Sun *et al.* 2019, 2020; Vani *et al.* 2019). Few studies have undertaken a detailed evaluation of the effect of Fusarium wilt on mungbean growth and yield production (Akhtar *et al.* 2005; Sandhu and Singh 2021), particularly within Australian production environments.

Overall, this study aims to dissect the relationship and behaviour of key agronomic traits contributing to yield under Fusarium wilt and disease-free environments. A diverse mungbean panel, consisting of several major commercial genotypes as well as genotypes available in germplasm collections, were evaluated in field trials in 2016 and 2021 under rainfed conditions, with and without *F. oxysporum*. Specifically, this study aims to explore: (i) the variation in key morphological and phenological traits underpinning yield within a diverse mungbean panel, and (ii) the effect of Fusarium wilt on traits underpinning yield. Improved understanding of how key agronomic traits influence yield is critical for developing new varieties with increased yield stability.

## Materials and Methods

### Field experiments

Field trials were sown in 2016 and 2021 at the Department of Agriculture and Fisheries (DAF) Hermitage Research Facility in Warwick, Queensland (QLD), Australia (28°12ʹ S, 152°5ʹ E) under rainfed conditions. A panel of 23 mungbean genotypes that represented a diverse range of phenological and morphological traits, as well as the black gram [*Vigna mungo*] cultivar, Onyx-AU, were characterized and sown in both trials (Supporting Information—Table S1). Accessions AGG 325964 (M10403), AGG 325968 (M12130), AGG 325973 (M11238), AGG 325977 (M08019), and AGG 325976 (Maus12-053) are co-owned by DAF and the Grains Research and Development Corporation (GRDC) all other accessions are available from the Australian Grains Genebank.

On 22 January 2016, the panel was sown in a paired plot row-column design with three replications (a total of six plots per genotype). Plots were 8 × 1.52 m containing 2 rows, with an interrow spacing of 0.76 m and plant density of 25 plants m^2^. On 28 January 2021, in a different experimental field block at the Hermitage Research Facility, the panel was sown in four replicates in a model-based row-column design using genetic relatedness, which was implemented using the R statistical package ‘od’ (Cullis *et al.* 2020) Plots were 5 × 1.52 m containing two rows, with an interrow spacing of 0.76 m and plant density of 25 plants m^2^. There was no previous record of *Fusarium* within the paddock prior to 2021. To provide an estimate of yield loss due to the disease in 2021, two check cultivars (Crystal and Celera II-AU) were sown in the adjacent field block that was not impacted by Fusarium wilt during the same season.

At the time of sowing, across both trials, seeds were inoculated with Group I inoculum (EasyRhiz™, New Edge Microbials Pty. Ltd) to ensure the establishment of rhizobia in root nodules to promote nitrogen fixation. Both trials had a basal application of Starter Z fertilizer at a rate of 25 kg ha^-1^. Weed, insect and disease controls were carried out as required to ensure adequate plant growth and development. At the 90 % black pod stage, the trials were desiccated with glyphosate and machine harvested to obtain grain yield data.

Daily meteorological data (minimum and maximum temperature, rainfall) were obtained from the Bureau of Meteorology weather station for Warwick (Station Number—041525) for the 2016 and 2021 seasons.

### Fusarium wilt assessment

During the 2016 trial, no Fusarium wilt symptoms were observed for the duration of the season. However, the 2021 trial experienced severe, widespread Fusarium wilt symptoms from early developmental stages (~25 DAS) (Supporting Information—Fig. S1). The presence of *Fusarium* occurred spontaneously via natural infection within the paddock and symptoms were present evenly throughout the paddock. A visual wilt score of 1–9 was given to all plots at 29 days after sowing (DAS), with 1 denoting no wilt symptoms and 9 denoting all plants in the plot had wilted due to Fusarium wilt.

A wilt tolerance index (WTI) was calculated for each genotype using their visual wilt score and final yield (t ha^-1^). The development of a weighted index is a common approach used to integrate multiple sources of data to estimate the overall merit or value of a breeding line. For disease selection, the establishment of an index is valuable due to multiple factors contributing to plant success under disease pressure (Forknall *et al.* 2019; Riaz *et al.* 2018). The wilt score was first normalized using the min–max standardization method, which is calculated as follows:


$$\begin{array}{l} x_{scaled}=\;\;\;\frac{\left(x\;-x_{min}\right)}{x_{max}-\;x_{min}}, \end{array}$$

where x is the trait value, $x_{min}$ is the minimum trait value across the data set and $x_{max}$ is the maximum trait value of the dataset. Normalized values were multiplied by their considered weighted value (yield 60 %; visual wilt score 40 %) and summed to form the tolerance index.

After identifying that the check cultivar in 2021, Celera II-AU, shared a similar yield in the non-*Fusarium* field and the 2016 trial, this cultivar was used as a benchmark within the season. This involved undertaking a least square means pairwise comparison with all genotypes in the 2021 disease trial against the Celera II-AU genotype in the trial. Based on the results of this test, we categorized genotypes into three groups, setting specific *P* value thresholds for significance. Genotypes with *P* values less than 0.001 were categorized as susceptible, those with *P* values ranging from 0.001 to 0.05 were considered moderately tolerant, and genotypes with *P* values greater than 0.05 were classified as tolerant. To further validate the differences between these three categories, an ANOVA was undertaken on the WTI categories to confirm the significant distinctions among the susceptible, moderately tolerant and tolerant genotypes.

### Confirmation of the disease-causing pathogen in the 2021 trial

To confirm that the presence of *F. oxysporum* was uniform across the field experiment, root samples were taken from plants randomly across the entire field experiment. Symptomatic stem and root tissues were surface sterilized in a 1.2 % (v/v) solution of NaOCl for 1 min and rinsed twice with distilled water. After drying on sterile blotting paper, several small pieces of tissue were cut from the advanced portion of the inner discoloured tissues of stem and root segments and placed onto the surface of water agar supplemented with streptomycin sulfate (100 mg L^−1^) in 9 cm diameter Petri plates. Plates were then incubated for 5 days under a 12 h 25 °C light/20 °C dark regime and then colonies were identified based on the protocol described by Leslie and Summerell (2006). Colonies identified as belonging in the genus *Fusarium* were sub-cultured onto carnation leaf agar and incubated under the same conditions for a further 7 days. Isolates were purified by transferring an agar block with a single germinating conidia onto fresh carnation leaf agar and potato dextrose agar plates. Cultures were incubated for 7–10 days under the same conditions then identified based on morphological characteristics (Leslie and Summerell 2006). All isolates identified as *F. oxysporum* based on morphological features were confirmed using DNA sequencing of the translation elongation factor (TEF1-*a*) gene region using the primers EF1—5ʹ-ATGGGTAAGGARGACAAGAC-3ʹ and EF2—5ʹ-GGARGTACCAGTSATCATGTT-3ʹ at the QLD Department of Agriculture and Fisheries plant pathology laboratory in Toowoomba, in accordance with Geiser *et al.* (2004).

### Phenology data collection

In both trials, all genotypes were monitored regularly to capture phenological development information, including days to 50 % flowering and 90 % black pod. Days to 50 % flowering was determined when 50 % of the plants in the plot had flowered. Days to 50 % flowering and 90 % black pod were used to quantify reproduction duration. Additional phenological information was captured during the 2021 trial including, days to first flower, flowering duration, days to mid-pod fill and days to the first black pod. Pod development duration was calculated using days to mid-pod fill and 90 % black pod. If genotypes did not flower by the end of the 2021 trial, a value of 85 days was assigned to denote the day at which the trial was desiccated for harvest.

### Morphology data collection

Biomass cuts (1.52 m^2^) were performed at two growth stages, 50 % flowering and 90 % black pod, from three replicates of all genotypes in 2016 and two replicates of all genotypes in 2021. The number of plants within each cut was recorded.

In 2016, the entire biomass cut at 50 % flowering was partitioned into stems and leaves, whereas the biomass cut performed at 90 % black pod was further partitioned into the following components: stems, leaves and reproductive components (pod clusters).

In 2021, biomass cuts performed at both growth stages were partitioned into a subset of 10 plants and separated into stems, leaves, flowers, immature pods, green pods and black pods. Green leaves were scanned with an LI-3100C Area Meter (LI-COR) to calculate total leaf area.

In both years, at the biomass cut performed at 90 % black pod, further pod dissection was undertaken to record number of pod clusters m^2^, total pod number m^2^, average pod length and average number of seeds pod.

Following dissection, all plant components were dried at 65 °C until consistent weight and weighed to obtain dry weight (DW). Plant component weight per m^2^ was calculated using biomass weights and plant number per biomass cut.

Grain yield was measured by harvesting the remaining plants within each plot with a plot harvester. The length and width of the plot were recorded prior to harvesting and used along with harvest grain to convert to tonnes per hectare. The 50 seed weight (50SW) was determined for each yield plot replicate.

Harvest index was calculated using the following formula:


$$\begin{array}{l} Harvest\;index=\;\frac{yield\;\;plant^{-1}}{total\;accumulated\;biomass\;plant^{-1}}. \end{array}$$

### Thermal time (TT) calculation

Daily thermal time (TT) was calculated for both experiments as described by Jones (1986) and Hammer *et al.* (1993) which used average temperature (*T*_a_) by applying a broken linear function of temperature (Equations (1)–(3)):


$$\begin{array}{l} TT=0,when\;T_{a}<T_{b}or\;T_{a}>T_{max}, \end{array}$$ (1)

$$\begin{array}{l} TT=T_{a}T_{b}when\;T_{b}<T_{a}<T_{opt}, \end{array}$$ (2)

$$\begin{array}{l} TT\;=\;(T_{opt}--\;T_{b})\;\left[1\;--\;\frac{T_{a}--\;T_{opt}}{T_{max}--\;T_{opt}}\right],\;when\;T_{opt}T_{a}T_{max}, \end{array}$$ (3)

where *T*_b_ denotes the base temperature of mungbean, *T*_opt_ denotes optimum temperature and *T*_max_ denotes maximum temperature, with values of 7.5 °C, 30 °C and 40 °C, respectively (Ellis *et al.* 1994). The TT at each phenological stage was determined by the accumulation of daily TT up until the respective development date.

### Statistical analysis

A linear mixed model was fitted in ASReml-R to estimate adjusted genotype means (best linear unbiased estimates; BLUEs) for all traits in each trial, where the plot trait observation and genotype were fitted as fixed effects and replicate number was fitted as a random effect. To test whether genotype was a significant effect on the trait within each year, the model was evaluated with a Wald chi-squared test. The adjusted means and standard error generated were used for further analyses and broad sense heritability was calculated for WTI. Differences in phenological and morphological means for each genotype across years were analysed using Welch’s *t*-tests. Pearson’s correlation coefficients among the trait means were also investigated to determine the strength of a linear association using the R package ‘corrplot’ (Wei *et al.* 2017).

## Results

### Comparison of meteorological conditions across field trials

**Figure 1.**
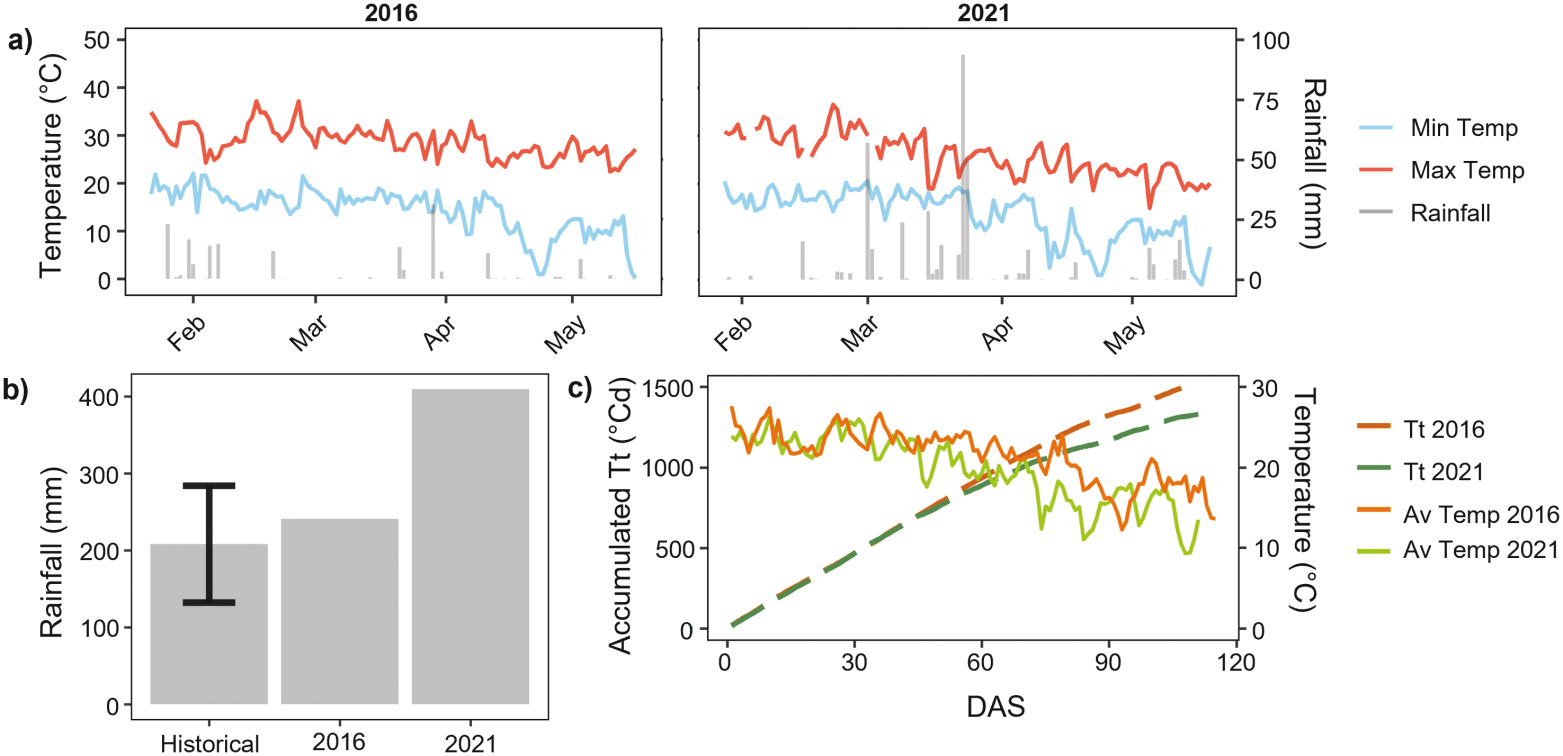
(**A)** Daily maximum temperature (red, °C), minimum temperature (blue, °C) and rainfall (grey, mm) data from sowing to harvest date for 2016 (left) and 2021 (right) trials at Warwick, Queensland, Australia, (B) Total rainfall obtained across 2016 and 2021 trials and historical site data (from 2000 to 2021) during January and May. Standard error denoted for historical data, (C) Cumulative thermal time (°Cd) plotted against days after sowing (DAS) for 2016 (orange dashed lines) and 2021 (green dashed lines) trials. Average temperatures (°C) plotted for 2016 (yellow), and 2021 (green) seasons.

Rainfall substantially differed between the two seasons (Fig. 1A), with 2021 receiving 406.6 mm of rain during the season compared to the 163.29 mm obtained during the 2016 trial (Fig. 1B). Comparing rainfall data at Warwick from 2000 to 2021, it was noted that the average rainfall Warwick typically received during the growth period (22 January to 19 May) was 208.33 mm (Fig. 1B). Therefore, rainfall received in 2016 can be described as average compared to the 2021 season which received substantially higher total rainfall compared to the historical mean.

Further, the pattern of rainfall events differed between the two seasons (Fig. 1A). Compared to 2016, there was a faster decline in temperature in March 2021, particularly maximum temperature (Fig. 1A) which led to differences in the rate of cumulative TT during the season. During February, the rate of cumulative TT was similar for both seasons (Fig. 1C) however, at approximately 47 DAS, cumulative TT diverged and began accumulating at a slower rate in 2021 than in 2016. From here forth, phenological developmental stages will be presented in cumulative TT.

### Fusarium wilt tolerance assessment in the 2021 trial

**Figure 2. (A):**
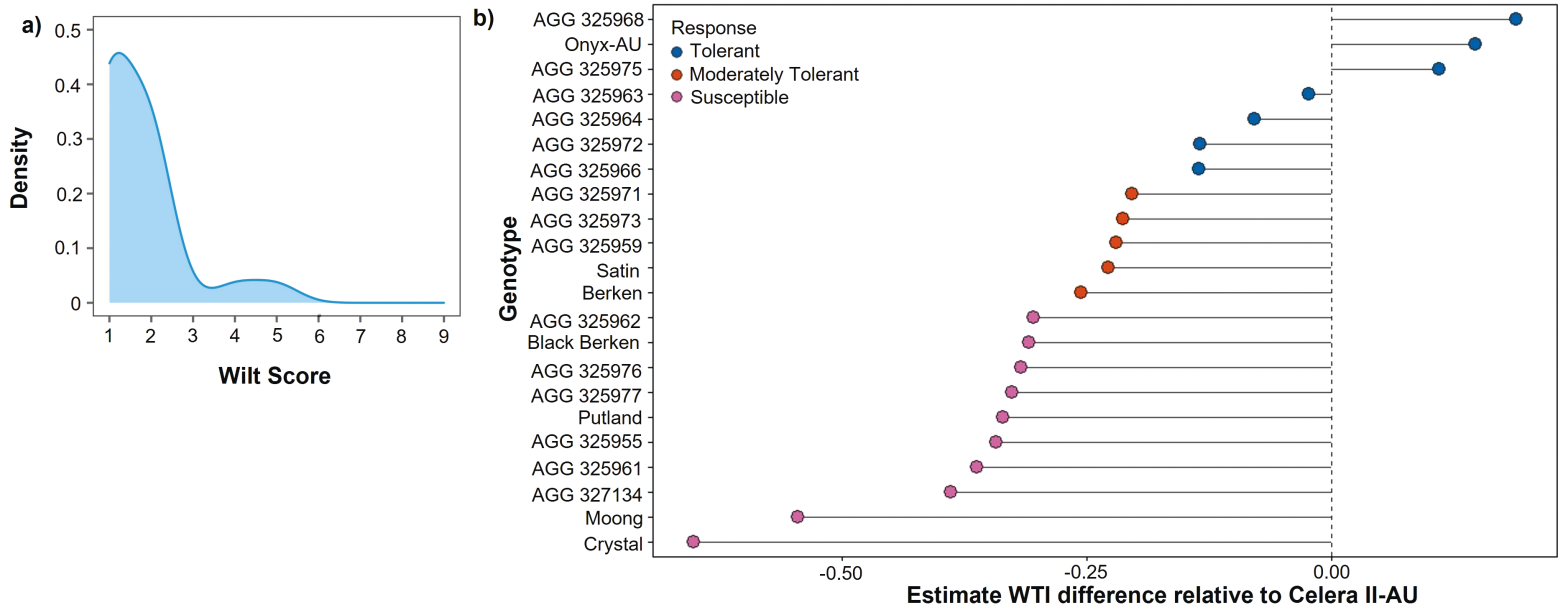
Density plot displaying a smoothed distribution of Fusarium wilt scores captured in the field at 29 DAS. (B) Graph of the estimated wilt tolerance index difference for the panel of genotypes evaluated in 2021, relative to Celera II-AU, which was identified to be a tolerant check. Significant differences between estimates were determined by using a least square means approach to undertake pairwise comparison between the check and all other genotypes. Based on the level of significant differences, genotypes were separated into three categories: susceptible (*P* ≤ 0.0001), moderately tolerant (P = 0.0001–0.019) and tolerant (*P* ≥ 0.02).

*Fusarium oxysporum* was confirmed in all symptomatic root samples collected from the Warwick field trial in 2021. For the initial *Fusarium* scores captured at 29 DAS, it was observed that the range of scores across genotypes ranged from 1 to 5 (Fig. 2A). No plots were observed to have all plants lost due to disease or significant damage (>7 score). When the WTI index was generated for all genotypes and compared to Celera II-AU, the genotypes were classified into three tolerance groups based on their significance; susceptible (*P* ≤ 0.001), moderately tolerant (*P* = 0.001–0.05) and tolerant (*P* ≥ 0.05, excluding AGG 325969 which had a significantly higher WTI then Celera II-AU and was therefore included in this group) (Fig. 2B). The heritability (h^2^) for the WTI was notably high, with a value of 0.94. Results from the ANOVA identified significant differences across these three groups (*P* ≤ 0.001). The cultivar Crystal was found to have the lowest WTI compared to accession AGG 325968 and black gram cultivar Onyx-AU which displayed the highest WTI. Further validation of the ranking of resistance and susceptibility for genotypes evaluated in this study was undertaken by comparing performance of concurrent genotypes grown in a breeding trial conducted by the National Mungbean Improvement Program where similar findings were made including that; Celera II-AU and AGG 325968 were determined to be tolerant to Fusarium wilt and Moong and Crystal determined to be susceptible (Supporting Information—Table S2).

### Variation and plasticity in key morphological and phenological traits underpinning yield

#### Phenological trait variation

**Figure 3.**
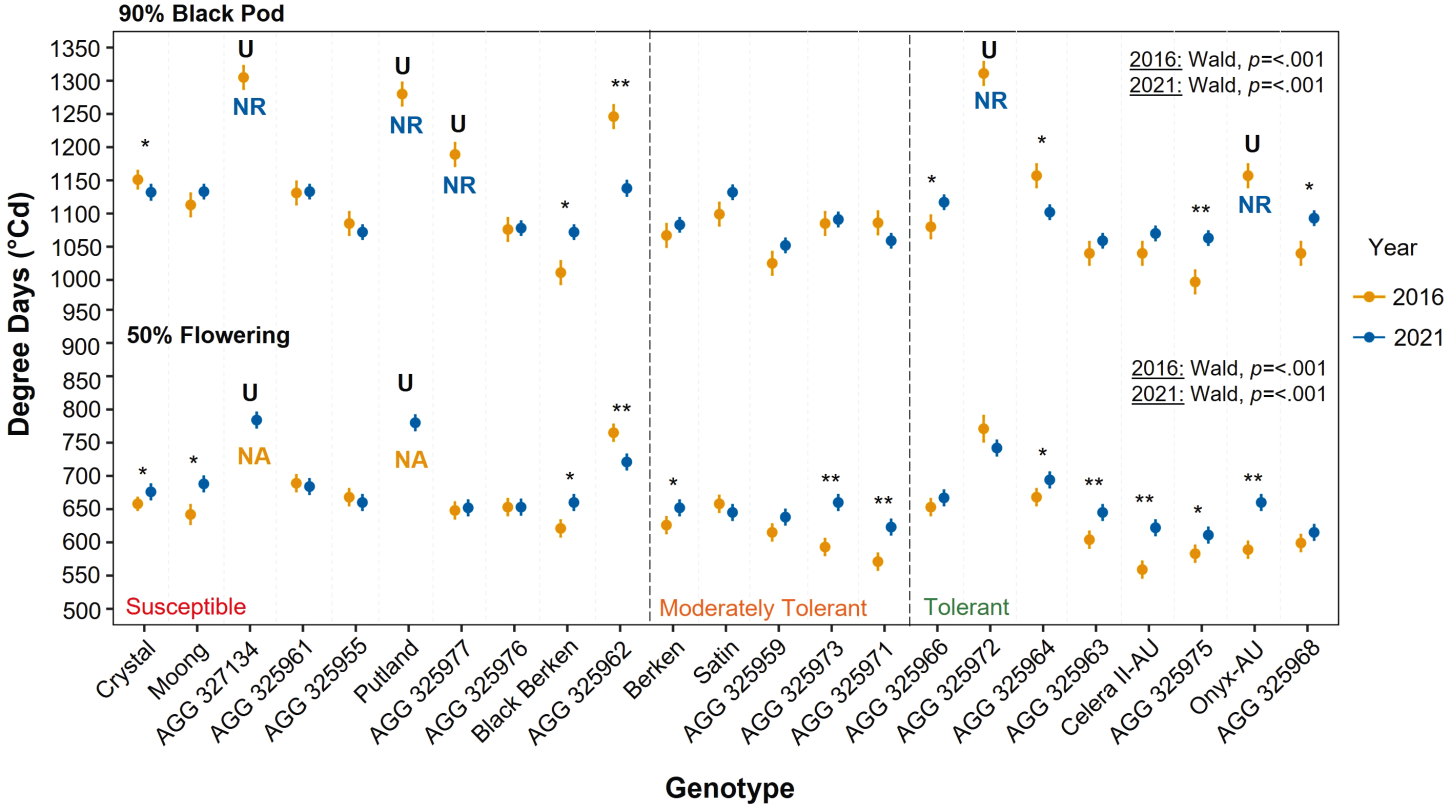
Genotype mean and standard error for TT to reach 50 % flowering and 90 % black pod in 2016 (yellow) and 2021 (blue). Significant differences between values across years were calculated by Welch Two Sample *t*-tests where the level of significance is denoted as *P < 0.05; **P < 0.01; ***P < 0.001. To test significant genotypic variance within each year, the *P* value from Wald Chi-Square tests are displayed on top right. NA notes data not available; NR notes genotype had not reached that developmental stage; U notes unable to perform test.

The majority of genotypes within the tolerant WTI group flowered significantly later in 2021, except for AGG 325966, AGG 325972 and AGG 325968, which flowered at similar times across both 2021 and 2016 (Fig. 3). Additionally, three genotypes from the moderately tolerant WTI group (Berken, AGG 325973 and AGG 325971), and from the susceptible WTI group (Crystal, Moong, Black Berken and AGG325962) also flowered significantly later in 2021 compared to 2016.

For 90 % black pod, most genotypes reached this stage at a similar TT across both the 2021 and 2016 seasons, with only three genotypes within the tolerant group and one within the susceptible WTI group reaching 90 % black pod significantly later in 2021 (Fig. 3). Despite flowering later in 2021, Crystal and AGG 325694 reached 90 % black pod significantly earlier in 2021 compared to 2016. AGG 325962 was observed to reach both 50 % flowering and 90 % black pod significantly earlier in 2021 compared to 2016.

For the additional traits captured in 2021, significant variation across the panel was observed (*P* ≤ 0.001) with values for TT to first flower trait ranging from 564 °C to 712 °Cd (Supporting Information—Fig. S2). There was a significant difference between the susceptible and tolerant WTI groups for this trait, with the tolerant group having significantly earlier flowering. For TT to mid pod fill and first black pod, there was also significant variation observed for each trait (*P* ≤ 0.001; 719 °C to 897 °C and *p* ≤ 0.001; 893 °C to 1119 °C, respectively), but there was no significant difference across WTI groups due to early, intermediate and late pod developing lines being present (TT to mid pod fill: *P* = 0.311 and TT to first black pod: *P* = 0.461). This trend was also noted for flowering and pod duration, with significant variation observed for the trait, but there being no significant differences across WTI groups due to the presence of short, intermediate and long flowering, and pod development genotypes within each group.

#### Morphological trait variation

Morphological traits varied significantly across both seasons for tolerant and susceptible groups, with no significant differences noted within the moderately tolerant group (Supporting Information—Fig. S3).

**Figure 4.**
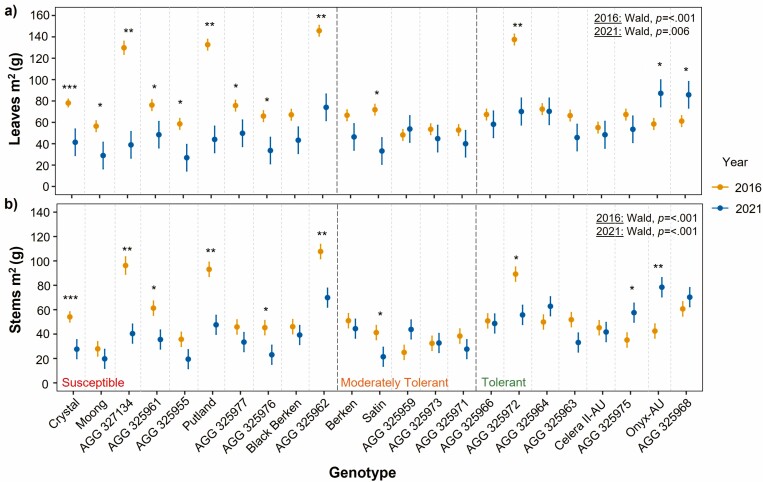
Genotype mean and standard error of DW (g) of (A) leaves m^2^ and (B) stems m^2^ at 50 % flowering in 2016 (yellow) and 2021 (blue). Significant differences between values across years weree calculated by Welch Two Sample *t*-tests. Level of significance *P < 0.05; **P < 0.01; ***P < 0.001. To test significant genotypic variance within each year, the *P* value from Wald Chi-Square tests is displayed on the top right.

At 50 % flowering, all genotypes within the susceptible WTI group, except for Black Berken, produced significantly less leaves m^2^ in 2021 compared to 2016 (Fig. 4A). This is similar to the findings for stems m^2^ within this group, except Black Berken, Moong, AGG325955 and AGG 325977 were not significantly different across seasons. In the moderately tolerant group, only Satin had significantly lower leaves m^2^ and stems m^2^ in 2021 as well as AGG 325972 in the tolerant group (Fig. 4B). Interestingly, Onyx-AU, AGG 325975 and AGG 325968 had either or both significantly higher leaves m^2^ and stems m^2^ in 2021 compared to 2016.

**Figure 5.**
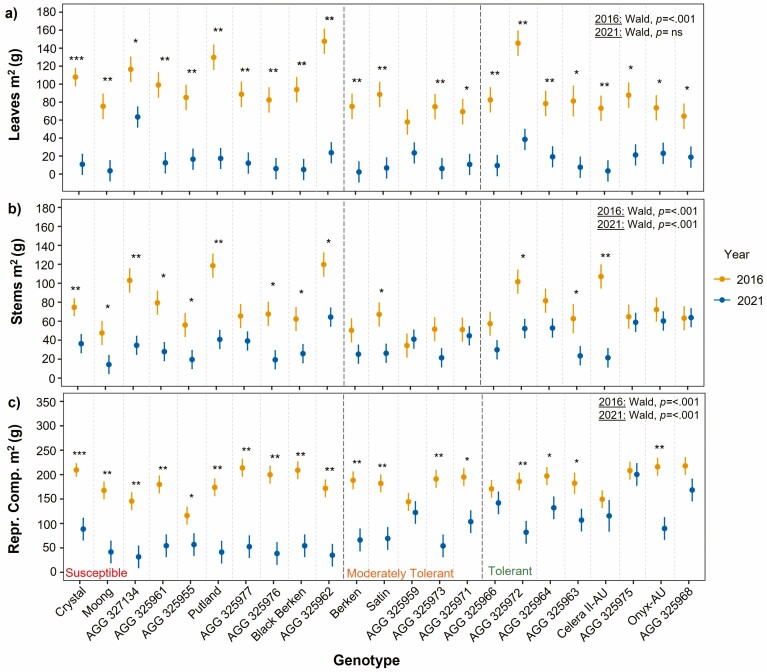
Genotype mean and standard error of DW (g) of (A) leaves m^2^, (B) stems m^2^ and (C) reproductive components m^2^ at 90 % black pod for genotypes across the 2016 (yellow) and 2021 (blue) seasons. Significant differences between values across years were calculated by Welch Two Sample *t*-tests. Level of significance *P < 0.05; **P < 0.01; ***P < 0.001. To test significant genotypic variance within each year, the *P* value from Wald Chi-Square tests is displayed on the top right.

At 90 % black pod, the majority of genotypes across all WTI groups had significantly less leaves m^2^ in 2021 compared to 2016 (Fig. 5A), except for Satin within the moderately tolerant group which was found not to have a significant difference across seasons. For the DW of stems m^2^ at this stage, there were fewer genotypes that had significantly different values across seasons (Fig. 5B). All genotypes within the susceptible group had significantly less DW of stems m^2^ in 2021 compared to 2016, while only one of the genotypes in the moderately tolerant group had significantly lower stems m^2^ in 2021 (Satin) and three genotypes within the tolerant group (AGG 325972, AGG 325963 and Celera II-AU). Most genotypes across all WTI groups had significantly lower DW of reproductive components m^2^, excluding AGG 325959 in the moderately tolerant group as well as Celera II-AU and AGG 325968 in the tolerant group that had no significant difference across years (Fig. 5C).

For the additional traits captured at 50 % flowering in the 2021 trial, significant variation was observed for the DW of flowers m^2^ (*P* ≤ 0.001; 0.58–5.17 g), green pods m^2^ (*P* ≤ 0.001; 0.02–2.43 g) and leaf area m^2^ (*P* ≤ 0.001; 719.18–40 033.29 cm^2^) (Supporting Information—Fig. S3). However, only leaf area was significantly different across WTI groups (*P* = 0.033), with the tolerant genotypes having higher leaf area compared to the susceptible genotypes. There were no significant differences between groups for flowers (*P* = 0.219) and green pods (*P* = 0.229).

#### Yield-related trait variation

**Figure 6.**
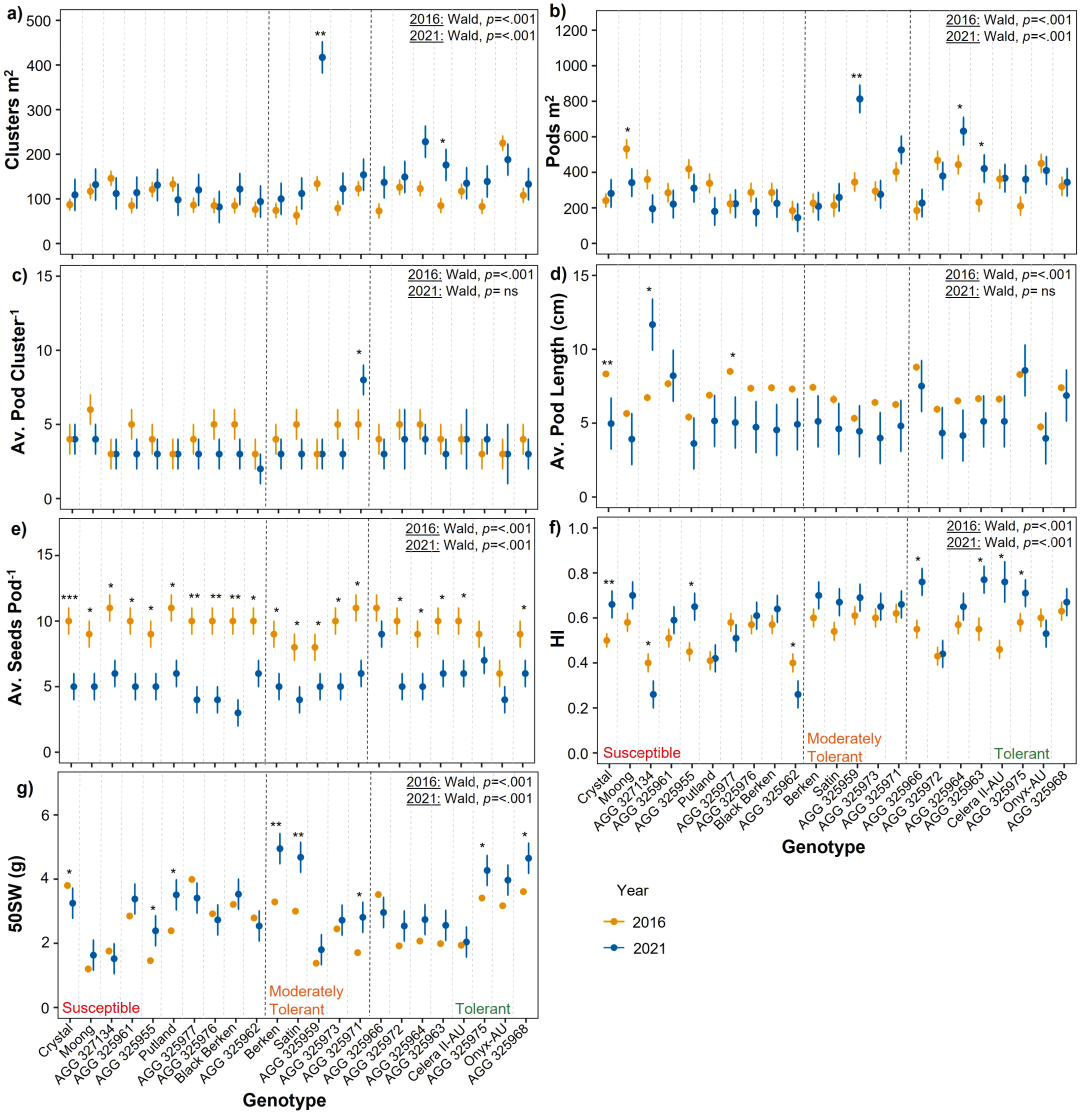
Genotype mean and standard error of yield components: (A) cluster m^2^, (B) pods m^2^, (C) average pods cluster^-1^, (D) average pod length, (E) average seeds pod^-1^, (F) HI and (G) 50SW for genotypes across 2016 (yellow) and 2021 (blue) seasons. Significant differences between values across years were calculated by Welch Two Sample *t*-tests. Level of significance *P < 0.05; **P < 0.01; ***P < 0.001. To test significant genotypic variance within each year, the *P* value from Wald Chi-Square tests are displayed on top right.

Significant genotypic variation was observed for most yield-related traits within 2021 and 2016 (Supporting Information—Fig. S4), excluding average pod cluster^−1^ and average pod length in 2021 (Fig. 6). Overall, most genotypes in 2021 were found to have similar clusters m^2^ as in 2016, with the exceptions of AGG 325959 and AGG 325963, which had significantly more clusters m^2^ in 2021 (between 91 and 283 more clusters m^2^) compared to 2016 (Fig. 6A). These genotypes also had significantly more pods m^2^ in addition to AGG 325964 in 2021. Although Moong within the susceptible group had significantly fewer pods m^2^ in 2021 (342) compared to 2016 (532) (Fig. 6B).

Notably, there were mostly no significant differences observed for genotypes across both seasons in regards to average pods cluster^−1^, excluding AGG 325971 which has significantly more pods cluster^−1^ in 2021 (Fig. 6C). When examining average pod length, Crystal and AGG 325977 within the susceptible group has significantly shorter pods in 2021 compared to 2016, whereas AGG 327134 had significantly longer pods in that season (Fig. 6D). There was also a significant reduction in the average number of seeds pod^−1^ in 2021 for all genotypes excluding AGG 325975 and black gram variety, Onyx-AU which there were no differences (Fig. 6E). Interestingly, the mean HI for most genotypes was slightly higher in 2021 than in 2016 (Fig. 6F). Crystal and AGG 325955 within the susceptible group, as well as AGG 325966, AGG 325963, Celera II-AU and AGG 325975 in the tolerant group had a significantly higher HI in 2021 compared to 2016. Susceptible genotypes, AGG 327134 and AGG 325962, had the opposite trend as they had significantly lower HI in 2021. Similarly, the mean 50SW of all genotypes in 2021 was slightly higher than in 2016 (Fig. 6G), genotypes AGG 325955, Putland, Berken, Satin, AGG 325971, AGG 325975 and AG 325968 having significantly higher 50SW in 2021 compared to 2016 whereas Crystal only had significantly lower 50SW in 2021 compared to 2016.

**Figure 7.**
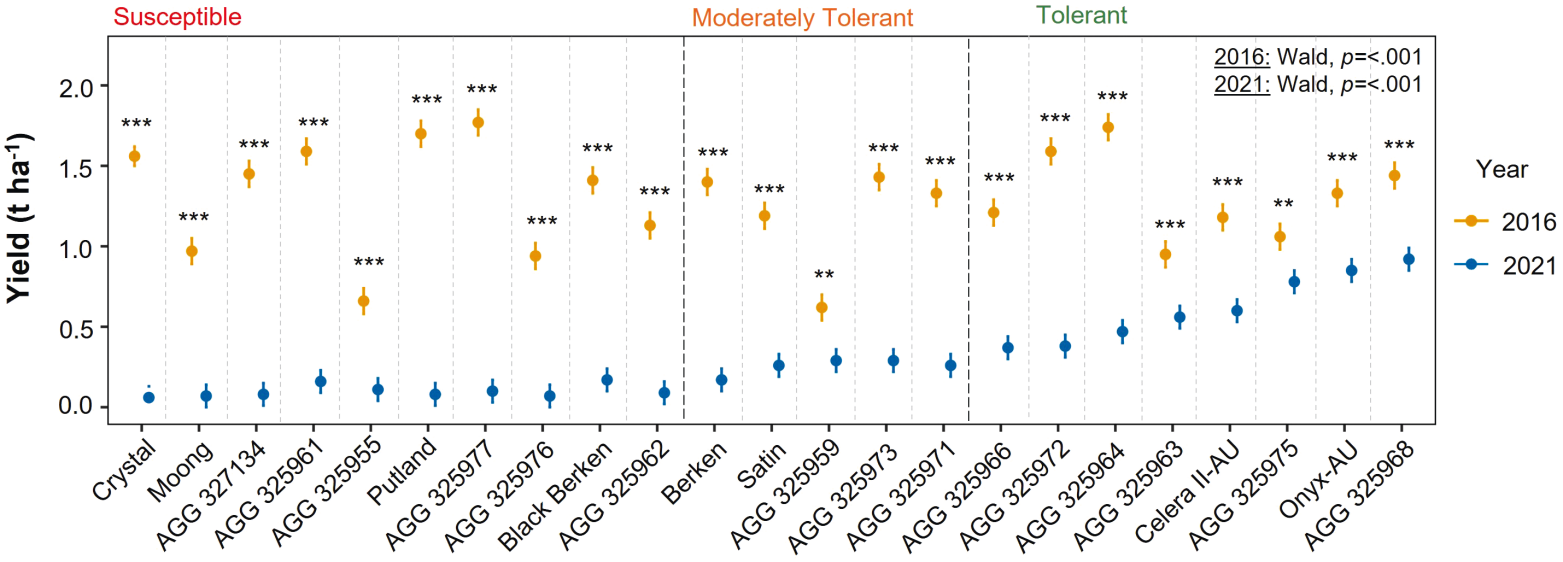
Genotype mean and standard error of yield (t ha^−1^) between panels in 2016 (yellow) and 2021 (blue). Significant differences between values across years were calculated by Welch Two Sample *t*-tests. Level of significance *P < 0.05; **P < 0.01; ***P < 0.001. To test significant genotypic variance within each year, the *P* value from Wald Chi-Square tests is displayed on the top right.

Yield was significantly lower in 2021 compared to 2016 for all genotypes (Fig. 7). The average yield reduction between 2021 and 2016 was 91.89 % for the susceptible group, 75.88 % for the moderately tolerant genotypes and 50.92 % for the tolerant genotypes. When comparing the two check cultivars in 2021 non-fusarium field compared to the 2016 field, a large yield reduction was noted for Crystal (64.74 %), whereas there was a slight increase in yield in the 2021 non-fusarium check trial (11.02 %). Comparing the two check cultivars that were grown adjacent to the 2021 trial and not impacted by Fusarium wilt, there was an 89.09 % yield reduction for Crystal (0.06 kg ha^−1^ and 0.55 kg ha^−1^, respectively) and 54.2 % reduction for Celera II-AU (0.61 kg ha^−1^ and 1.31 kg ha^−1^, respectively).

### Relationships between *Fusarium* and phenology, morphology and yield-related traits

**Figure 8.**
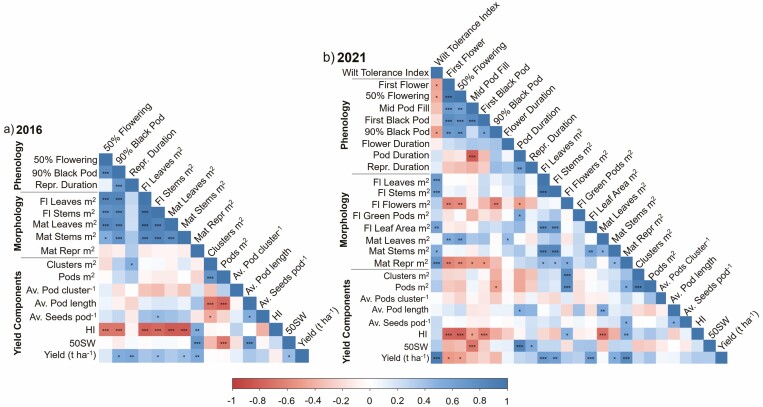
Correlation coefficient matrix showing associations of phenological, morphological and yield component traits among diverse mungbean panels evaluated in (A) 2016 and (B) 2021. Colour key depicting Pearson’s correlation coefficients (*r*). Level of significance *P < 0.05; **P < 0.01; ***P < 0.001.

Among the 16 traits captured across both seasons, the DW of stems m^2^ at 50 % flowering as well as stems m^2^ and reproductive components m^2^ at 90 % black pod were positively correlated to yield in 2021 and 2016 (Fig. 8). Furthermore, the DW of leaves m^2^ at both time points demonstrated a significantly positive relationship with yield in 2021, as well as TT to reach 90 % black pod, reproductive duration and 50SW in 2016. For the additional traits measured in 2021, leaf area m^2^ and the WTI had a significant positive relationship with yield. Notably, TT to first flower had a negative correlation with yield in 2021. WTI was further observed to be negatively correlated with TT to reach first flower, 50 % flowering and 90 % black pod. WTI did, however, have a significant positive relationship with the DW of leaves m^2^, stems m^2^, leaf area m^2^ at 50 % flowering as well as DW of stems m^2^ and reproductive components m^2^ at 90 % black pod.

Further, in the 2016 season, 50SW was positively correlated with average pod length, however, a negative relationship was observed with pods m^2^. Therefore, large-seeded genotypes produced less pods m^2^, but these pods were longer and had more average seeds pod^−1^, leading to higher yield.

## Discussion

Field trials using the same germplasm set were conducted in two seasons with absence (2016) and presence (2021) of Fusarium wilt. This has enabled a detailed dissection on the effect of Fusarium wilt on the development and production of yield which, to our knowledge, has not been undertaken on mungbean previously. Among the various traits studied, early biomass development emerged as a pivotal factor driving yield in both the presence and absence of disease, although it was significantly influenced by the presence of Fusarium wilt. This study also identified several tolerant genotypes that exhibit different tolerance mechanisms to Fusarium wilt with regard to flowering time and the accumulation and maintenance of early biomass.

### Traits associated with yield in the presence and absence of Fusarium wilt

There are several shared traits associated with yield across environments with the presence and absence of disease. These traits include stem biomass accumulated at 50 % flowering and 90 % black pod as well as the DW of reproductive components at 90 % black pod, which could be indicative of the plant being able to accumulate enough biomass to support reproductive components (Fig. 8). HI was also positively correlated to DW of reproductive components that demonstrates the importance of partitioning carbohydrates for yield. Importantly, HI was negatively correlated to flowering time (TT to 50 % flowering) across the two seasons, which may be a consequence of establishing a sufficiently large reproductive sink to transition from vegetative to reproductive growth (Geetika *et al.* 2022).

In the absence of disease, yield is driven by several phenological and morphological traits. For instance, in the 2016 trial, large-seeded genotypes had higher yields and fewer pods m^2^ and clusters m^2^ (Fig. 8A). Most of these findings are supported by previous mungbean yield evaluation studies conducted using Indian genotypes (Mondal *et al.* 2011, 2012), except for seed size which has often been reported to have both a positive and negative association with yield depending on the genotype and environment (Rao *et al.* 2006; Hakim 2008; Mondal *et al.* 2011). These results suggest that there is a compensatory mechanism between the number of clusters and pods, as well as pod length and seed size due to either competition for assimilates between sinks or competition between the different source components (i.e. leaves, stems and roots), which has been reported previously (Clifford 1979; Rao and Ghildiyal 1985). There is an opportunity to explore potential trait combinations such as cluster m^2^, pods m^2^, average pod length and average seeds pod^−1^ to improve overall productivity.

### Fusarium wilt constrains yield by impacting source availability

Under disease conditions, a reduction in aboveground biomass was predominantly observed in susceptible genotypes, leading to a corresponding reduction in yield. This was particularly evident for canopy biomass accumulated before flowering, with the genotypes in the susceptible group accumulating significantly less leaves m^2^ at this stage (Fig. 2A). Reductions in biomass are likely a consequence of the infection and growth cycle of *F. oxysporum* which damages the vascular tissue, resulting in leaf chlorosis, an interruption to carbohydrate assimilation and, therefore, reduced assimilate supply (Wang *et al.* 2013; Li *et al.* 2017) and stunted growth (Cachinero *et al.* 2002; Hao *et al.* 2009). The importance of maintaining high leaf biomass was evident in the presence of Fusarium wilt with a significant positive relationship with DW of reproductive components and yield not observed in absence of disease in 2016 (Fig. 8B). Overall, reductions in aboveground biomass at the 50 % flowering stage led to a 41–96 % yield loss.

A further impact of Fusarium wilt was a reduction in reproductive components including pod length and seeds per pod despite the increase in pods per m^2^. This may be a consequence of decreased pod filling due to decreased assimilate supply and/or pinching (data not presented). Similar trends were reported in Canola infected with *Fusarium*, whereby plants infected with the disease had smaller pods that had no seeds (Kochman 2007).

Fusarium wilt leads to a progression of symptoms in crops (Di *et al.* 2016; Kelly 2018) As a result, some genotypes that were given an initial disease score of 1 (no symptoms) were later determined to be susceptible or moderately tolerant based on their WTI scores. This highlights the severe progression of *Fusarium* over time and suggests the limitation of scoring disease at a single time point as typically occurs in plant breeding programmes (Munawar *et al.* 2011; Tian *et al.* 2022). Due to the importance of aboveground biomass at the flowering stage, scoring for tolerance to *Fusarium* would be appropriate at this timepoint. Furthermore, the implementation of high-throughput phenotyping platforms such as unmanned aerial vehicles, which rapidly monitor canopy biomass would allow for efficient quantification of disease impacts over development (Van Haeften *et al.* 2023).

#### Opportunity to exploit tolerant mungbean accessions for breeding

From the 23 genotypes investigated in this study, 8 genotypes were found to be tolerant to Fusarium wilt based on their WTI. Overall, six of the tolerant genotypes flowered later in 2021 (under the presence of disease) compared to 2016. Previous findings in Arabidopsis and sorghum determined that Fusarium wilt accelerates the transition to flowering and that later flowering lines were associated with tolerance (Lyons *et al.* 2015; Mrema *et al.* 2020). The identification of tolerant mungbean lines in this study with both early and late flowering time suggests that phenology and tolerance may be uncoupled. Similar findings were reported in chickpea as tolerant lines were clustered into four categories differing in early and late flowering lines with high and low 100 seed weight (Halila and Strange 1997).

Seven of the eight tolerant mungbean lines maintained or increased aboveground biomass (stems, leaf) at 50 % flowering. This further reinforces the importance of aboveground biomass to support yield development in mungbean. However, a significant reduction in aboveground biomass was observed for AGG 325972 which suggests that multiple mechanisms for tolerance may be present. Interestingly all tolerant genotypes, excluding Onyx-AU (*Vigna mungo*), had a high HI demonstrating effective remobilization of carbohydrate from source to sink.

A number of plant defence mechanisms against Fusarium wilt have been investigated in several crops including soybean (Lanubile *et al.* 2015) and common bean (Xue *et al.* 2015). These studies have found that after infection many *Fusarium* resistant genotypes contain genes that contribute to R-gene mediated defence, structural defence, signal transduction and secondary metabolites (Upasani *et al.* 2017; Chen *et al.* 2019) and these molecular mechanisms have also recently been confirmed in resistant mungbean genotypes (Chang *et al.* 2021). Further research is required to determine the mechanisms underpinning disease tolerance in the mungbean genotypes identified in this study. These genotypes represent a valuable source of donor materials that could be incorporated into breeding programmes and for population development. Utilizing a range of tolerant lines that exhibit varying phenological and morphological traits could allow breeders to create superior lines that are adapted to different production environments.

## Conclusion

The narrow genetic background of mungbean has created vulnerability to a range of biotic stresses, including Fusarium wilt, which can lead to devastating yield losses for growers. In this study, we examine how this pathogen impacts mungbean growth and identified tolerant genotypes and potential traits of interest to improve yield in environments that are not affected by Fusarium wilt. In particular, the important role of biomass accumulation prior to the mid-pod fill stage is confirmed as underpinning yield development in mungbean. Further studies on these less susceptible genotypes to dissect the genes associated with Fusarium wilt tolerance are required to identify traits of interest for incorporation in mungbean breeding programs.

## Sources of Funding

This research was funded by Grains Research and Development Corporation (GRDC; UOQ2101-003RSX) and the International Mungbean Improvement Network (ACIAR; CIM2014/079).

## Supplementary Material

plae021_suppl_Supplementary_Tables_S1-S2_Figures_S1-S4

## Data Availability

This dataset is available at UQ eSpace https://doi.org/10.48610/39782c7.
