# Supplementary material for: Fusarium wilt constrains mungbean yield due to reduction in source availability
Source: AoB Plants. 2024 Apr 9;16(2):plae021. doi: 10.1093/aobpla/plae021 (PMC11034375; doi:10.1093/aobpla/plae021)
Supplement: plae021_suppl_Supplementary_Tables_S1-S2_Figures_S1-S4 [file plae021_suppl_supplementary_tables_s1-s2_figures_s1-s4.pdf]

Supplemental Table S1. *Mungbean genotypes evaluated in this study. The specific genotype name bolded will be used throughout the study. Descriptions are based on information available from the Australian Grains Genebank and the Department of Agriculture and Fisheries.*

ACCESSION ID[1]	ALTERNATIVE NAME	ORIGIN	TYPE	SEED SIZE	SEED COLOUR
**AGG 325975**	AusTRC 321818	Malaysia	Accession	Large	Green
**AGG 325971**	AusTRC 324134	Taiwan	Accession	Small	Green
**AGG 325972**	AusTRC 324277	India	Breeding line	Small	Green
**AGG 325961**	AusTRC 324363	Taiwan	Breeding line	Large	Green
AGG 325978	**Berken**	Philippines	Cultivar	Large	Green
AGG 325970	**Black Berken**	Australia	Cultivar	Large	Black
AGG 325984	**Celera II-AU**	Australia	Cultivar	Small	Green
**AGG 325966**	CHIH-CO	Taiwan	Accession	Large	Green
**AGG 327134**	CPI30757	Myanmar	Accession	Small	Green
**AGG 325955**	CPI62672	Afghanistan	Accession	Small	Yellow
**AGG 325959**	CPI62822	Iran	Accession	Small	Speckled
AGG 325979	**Crystal**	Australia	Cultivar	Large	Green
**AGG 325962**	EJP2	Australia	Breeding line	Medium	Yellow
**AGG 325977**	M08019	Australia	Breeding line	Large	Green
**AGG 325964**	M10403	Australia	Breeding line	Large	Green
**AGG 325973**	M11238	Australia	Breeding line	Small	Green
**AGG 325968**	M12130	Australia	Breeding line	Large	Green
**AGG 325963**	M773	Australia	Breeding line	Small	Green
**AGG 325976**	Maus12-053	Australia	Breeding line	Large	Green
AGG 325960	**Moong**	India	Cultivar	Small	Yellow
AGG 329004	**Onyx-Au *(Blackgram)***	Australia	Cultivar	Large	Black
AGG 325945	**Putland**	Australia	Cultivar	Medium	Green
AGG 325969	**Satin**	Australia	Cultivar	Large	Green

[1]*Accession ID based on records from Australian Grains Genebank*

Supplemental Table S2. *Best linear unbiased estimates (BLUEs) for fusarium wilt scores of key mungbean genotypes evaluated in a breeding trial at Department of Agriculture and Fisheries (DAF) Hermitage Research Facility in Warwick, Queensland (QLD), Australia (28°12′ S, 152°5′ E)* in 2023 *(unpublished). A visual wilt score of 1-9 was given to all plots at 70 days after sowing (DAS), with 1 denoting no wilt symptoms and 9 denoting all plants in the plot had wilted due to Fusarium wilt.*

GENOTYPE	FUSARIUM SEVERITY SCORE (BLUE)
**AGG 325968**	3
**Celera II-AU**	3
**Moong**	9
**Crystal**	9

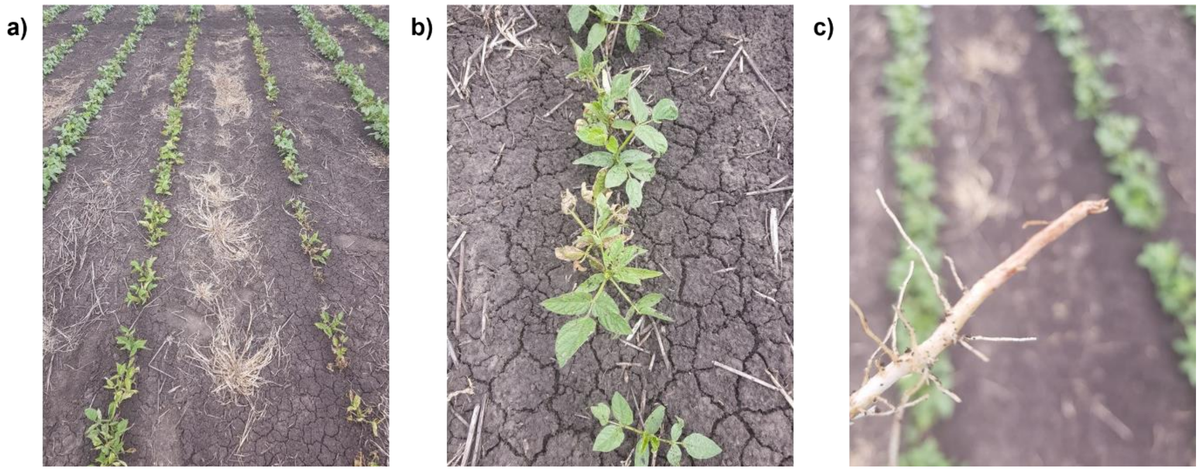

Supplemental Figure S1. a) *Example of plot infected with Fusarium****. b)*** *Example of foliar damage of Fusarium infected plants****, c)*** *Example of root system of Fusarium infected plant*

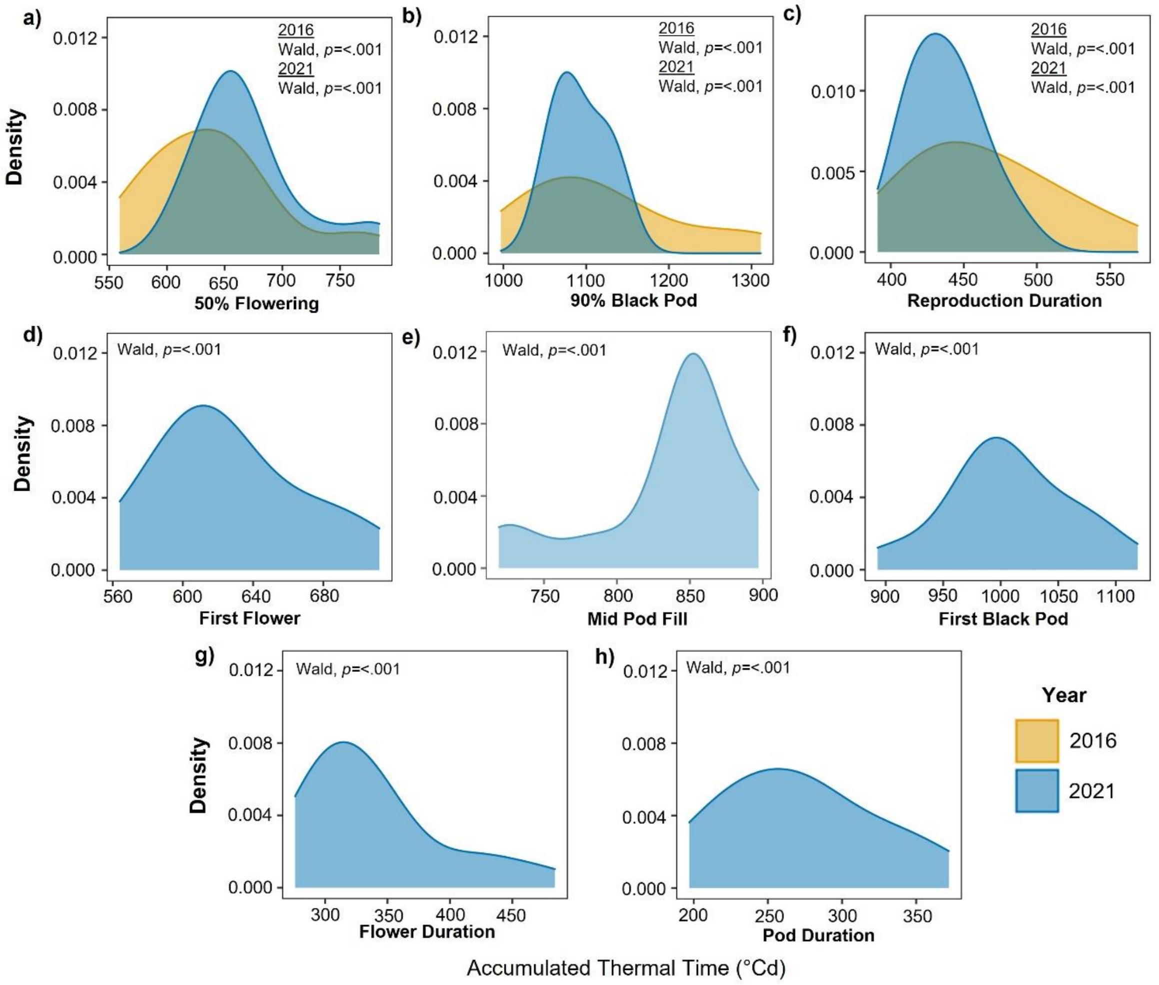

Supplemental Figure S2. a-h) *Density plots displaying a smoothed distribution of phenological traits at* ***(a)*** *50% Flowering,* ***(b)*** *90% Black Pod,* ***(c)*** *Reproduction Duration,* ***(d)****First Flower,* ***(e)*** *Mid Pod Fill,* ***(f)*** *First Black Pod,* ***(g)*** *Flower Duration and* ***(h)*** *Pod Duration for diverse mungbean panel in 2016 (yellow) and 2021 (blue) season. To test significant genotypic variance in each year, the P value from Wald-chi Squared Tests are displayed.*

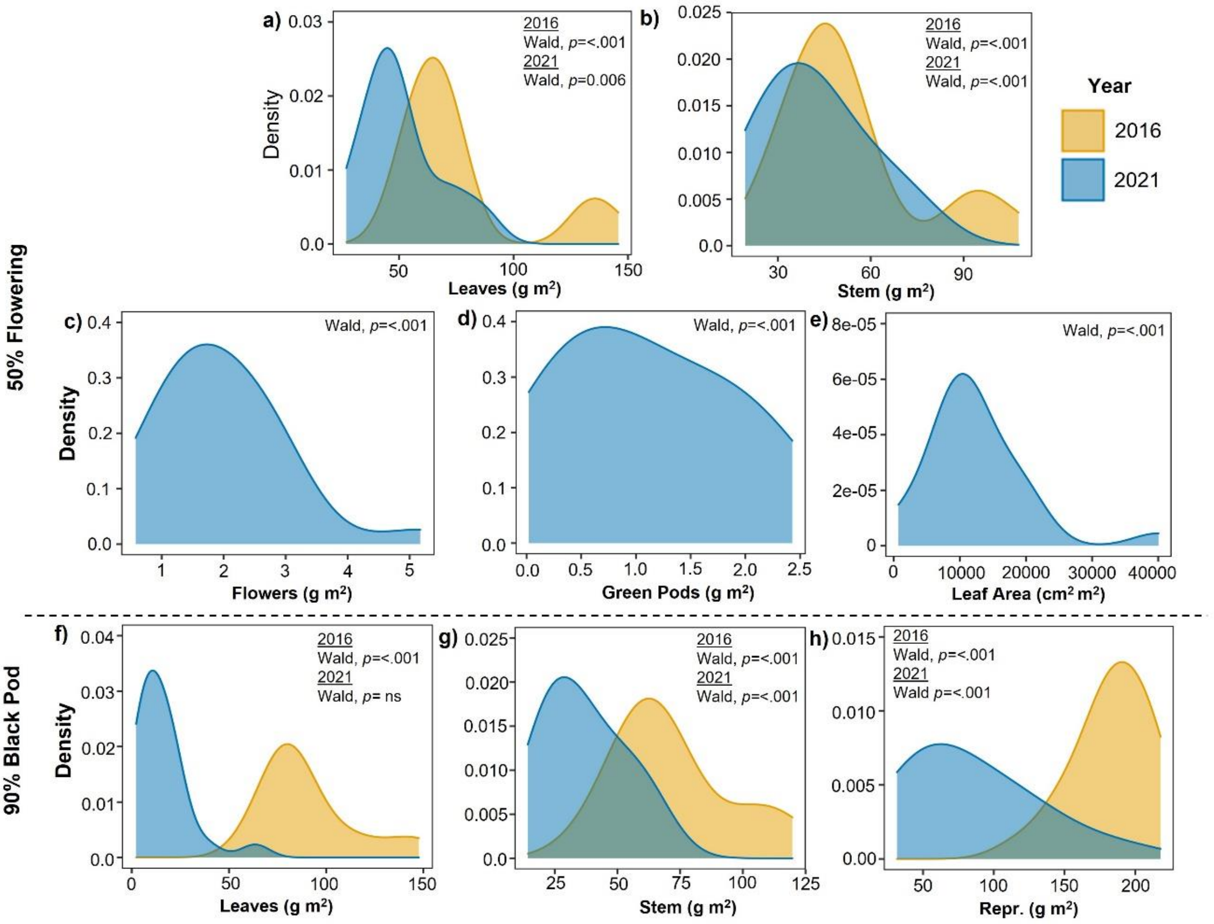

***Supplemental Figure S3. a-h)** Density plots showing distribution of morphological traits at 50% flowering and 90% black pod in diverse mungbean panel in 2016 (yellow) and 2021 (blue). **(a)** Leaves (g m^2)**, (b)** stems (g m^2), **(c)** Flowers (g m^2), **(d)** Green pods (g m^2), **(e)** Leaf area (cm^2 m^2), **(f)** Leaves (g m^2), **(g)** Stem (g m^2) and **(h)** Reproductive components (g m^2). To test significant genotypic variance in each year, the P value from Wald-chi Squared Tests are displayed.*

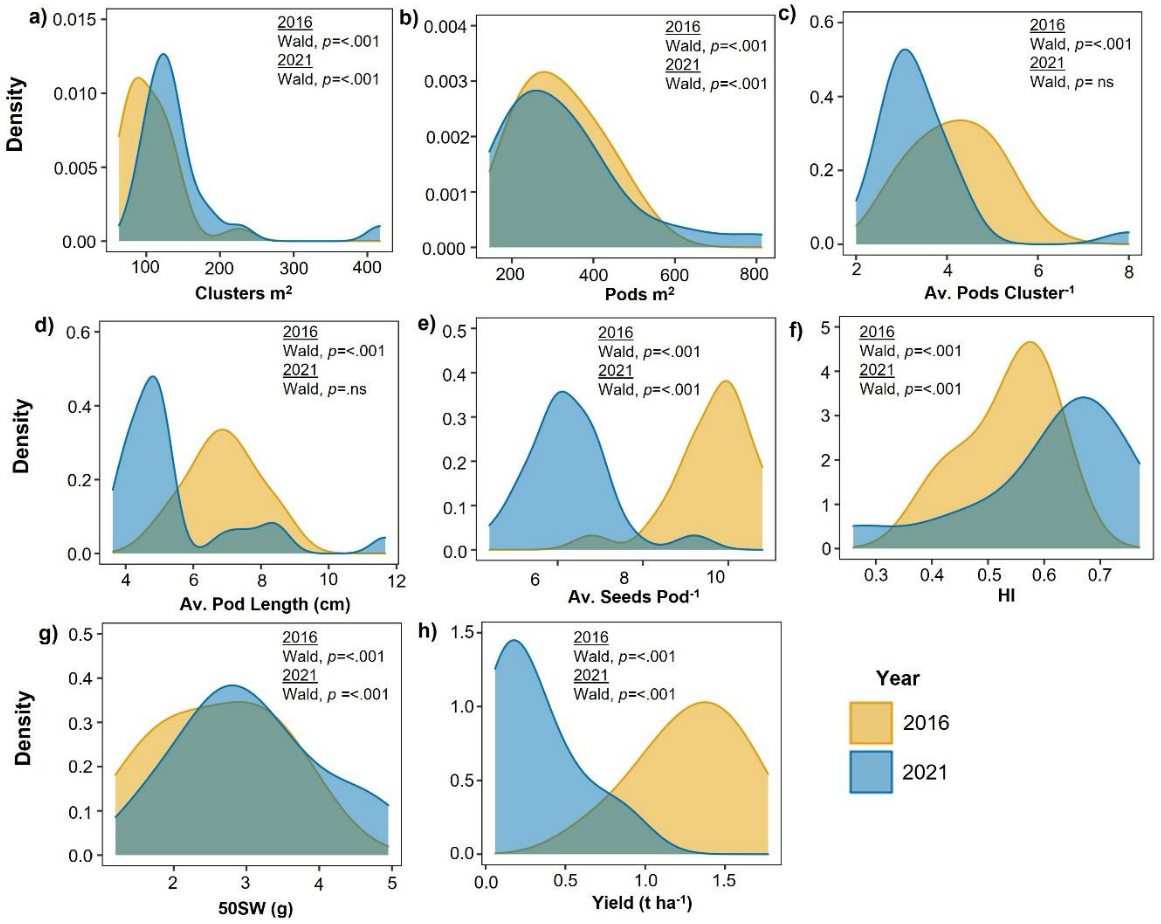

Supplemental Figure S4. a-h) *Density plots showing distribution of yield component traits in diverse mungbean panel across 2016 (yellow) and 2021 (blue) season.* ***(a)*** *Clusters m*^{2}***, (b)*** *Pods m*^{2}*,* ***(c)*** *Average pods cluster*$^{-1}$*,* ***(d)*** *Average pod length (cm),* ***(e)*** *Average seeds pod*$^{-1}$*,* ***(f)*** *HI,* ***(g)*** *50SW (g),* ***(h)*** *yield (t ha*$^{-1}$*). To test significant genotypic variance in each year, the P value from Wald-chi Squared Tests are displayed.*
